# Multi-Scale Depthwise Separable Capsule Network for hyperspectral image classification

**DOI:** 10.1371/journal.pone.0308789

**Published:** 2024-08-28

**Authors:** Lin Wei, Haoxiang Ran, Yuping Yin, Huihan Yang

**Affiliations:** 1 School of Software, Liaoning Technical University, Huludao, Liaoning, China; 2 The Department of Basic Education, Liaoning Technical University, Huludao, Liaoning, China; 3 Faculty of Electrical and Control Engineering, Liaoning Technical University, Huludao, Liaoning, China; 4 School of Electronic and Information Engineering, Liaoning Technical University, Huludao, Liaoning, China; Manipal Institute of Technology, INDIA

## Abstract

Addressing the challenges in effectively extracting multi-scale features and preserving pose information during hyperspectral image (HSI) classification, a Multi-Scale Depthwise Separable Capsule Network (MDSC-Net) is proposed in this article for HSI classification. Initially, hierarchical features are extracted by MDSC-Net through the employment of parallel multi-scale convolutional kernels, while computational complexity is reduced via depthwise separable convolutions, thus reducing the overall computational load and achieving efficient feature extraction. Subsequently, to enhance the translational invariance of features and reduce the loss of pose information, features of various scales are processed in parallel by independent capsule networks, with improvements in max pooling achieved through dynamic routing. Lastly, features of different scales are concatenated and integrated through the concatenate operation, thereby facilitating precise analysis of multi-level information in the hyperspectral image classification process. Experimental comparisons demonstrate that MDSC-Net achieves average accuracies of 94%, 98%, and 99% on the Kennedy Space Center, University of Pavia, and Salinas datasets, respectively, indicating a significant performance advantage over recent HSI classification models and validating the effectiveness of the proposed model.

## 1 Introduction

Hyperspectral imaging (HSI), as a remote sensing data source rich in physical and chemical properties, has demonstrated immense potential for applications in fields such as earth science, resource exploration, and environmental monitoring [1–3]. In these applications, the task of HSI classification fundamentally involves the precise identification of land cover based on the spectral characteristics of pixels, thereby aiding scientific research and decision-making. The classification of HSI, especially its feature extraction, occupies a crucial position in the domains of machine learning and image analysis, and has long been a subject of keen interest among researchers. Although this task poses significant challenges for machines, precise labels can be created through manual annotation of images, providing high-quality training data for supervised learning and thereby effectively supporting the learning process. I In the field of feature extraction, the main approaches to addressing the problem are divided into two categories: one is based on pixels, and the other is based on regions. In pixel-based classification methods, each pixel is classified individually, primarily based on its independent spectral information, without consideration of its spatial relationship with surrounding pixels [4]. Although straightforward, this approach has clear limitations as it neglects the potential spatial relationships between pixels. Conversely, a more efficient method involves analyzing the data cube as a whole, thereby leveraging both spatial and spectral information [5]. This approach more comprehensively considers the spatial connections between pixels, offering possibilities for more accurate image analysis. Hence, this study focuses on region-based analysis methods.

Neural networks, particularly Convolutional Neural Networks (CNN) that have been developed since the 1970s, have shown significant effectiveness in addressing such issues [6]. Architectures like AlexNet [7], ResNet [8], and GoogLeNet [9], although originally designed for processing the red, green, and blue (RGB) bands in visible light, are equally applicable and effective for multi-channel data analysis. With the widespread application of CNN in image processing, their outstanding performance in HSI classification tasks has been confirmed by numerous studies [10–12]. For instance, 3D-CNN have been used for feature extraction from HSI [13]; the introduction of attention mechanisms [14] has optimized feature map extraction; and network structures like HybridSN [15] and SpectralNET [16] have achieved breakthroughs in HSI classification accuracy. Notably, recent studies [17, 18] have further proposed various methods to enhance HSI classification accuracy. The aforementioned research, although achieving significant accomplishments in HSI classification tasks, still faces limitations in aspects such as the reduction of spatial information due to pooling operations, and large model parameter size. Notably, CNN struggle to effectively recognize object pose information, as their convolutional filters cannot represent feature transformation activities. To overcome the limitations of traditional CNN approaches, the concept of capsule networks was first introduced by Hinton and colleagues [19]. Capsules are groups of neurons that describe an entity’s pose and probability of existence, containing more information about the entity’s attributes than scalar neurons in CNN. The capsule network (CapsNet), proposed by Sabour and others [20], encodes the probability of an object’s presence and its pose through the length and orientation of activity vectors, enhancing the performance of capsule networks in image analysis. Further, H. Zhang and colleagues applied capsule networks to HSI classification [21], achieving significant results. Additionally, Hinton and colleagues proposed matrix capsules with EM routing [22], addressing some deficiencies of the dynamic routing algorithm in [20], such as using the negative log variance of Gaussian clusters to measure the consistency between pose vectors, and representing poses with matrices instead of vector lengths.

Recently, the trend has been to apply capsule network technology to HSI analysis. Methods based on capsule networks have demonstrated significant effectiveness in extracting deep semantic features of hyperspectral images. This approach utilizes neurons within capsule networks to simultaneously capture both the spectral and spatial features of HSI images, as evidenced by several studies [23–25]. Although existing capsule networks have improved some aspects of CNN in HSI classification tasks, they face significant challenges in parsing complex spectral-spatial features that vary with scale. Particularly in capturing and analyzing multi-scale features, capsule networks often fail to fully extract spectral characteristics, thereby affecting the effective interpretation of rich geophysical details and multi-level information in HSI, which in turn reduces the accuracy of the classification process. In summary, the problem this study aims to address in HSI classification tasks is that the pooling operations in convolutional networks often lead to a reduction in spatial information, thereby diminishing the ability to effectively recognize object pose information [26]. Additionally, capsule networks have not been sufficiently capable of extracting multi-scale spectral features [27]. To overcome the limitations of existing methods, we propose an innovative Multi-Scale Depthwise Separable Capsule Network (MDSC-Net). In summary, the main contributions of this paper are as follows:

1. A novel multi-scale capsule network architecture is introduced, utilizing multi-scale convolutional kernels to extract features at different levels, effectively parsing the rich detail and multi-layered information in hyperspectral images. This enhances the precise identification and understanding of complex terrains and land cover types. Feature maps at each scale are processed independently by capsule networks to accurately capture subtle changes in land cover at different scales, ensuring the integrity of spatial information and improving the efficiency of hyperspectral image data analysis.
2. By employing a dynamic routing mechanism instead of the traditional max-pooling method, the dimensionality of feature maps is effectively reduced, and the pose matrices in capsule networks enhance the model’s translation invariance of features. This approach improves the network’s ability to capture details when handling complex terrains and ensures the in-depth analysis of the rich hierarchical information in hyperspectral image data, achieving highly accurate and efficient data processing.
3. Depthwise separable convolution is incorporated into the architecture by replacing the original three-dimensional convolution structure with depthwise and pointwise convolutions. This ensures efficient feature extraction while significantly reducing the model’s computational complexity, thereby effectively alleviating the overall computational burden.
4. In addition to evaluating the effectiveness of the proposed MDSC-Net architecture in terms of overall accuracy (OA), average accuracy (AA), and kappa coefficient (Kappa), this method is also compared with existing state-of-the-art hyperspectral image classification methods in terms of efficiency.

## 2 Basic principles of the EM routing algorithm

The EM routing algorithm plays a crucial role in selecting the optimal capsule pathways to optimize information flow within this model. The algorithm involves M-steps and E-steps. The E-step equation is as follows:

$$Q(\theta |\theta^{t})=E[\log L(\theta |Z)|X,\theta^{t}]$$ (1)

Where *θ* represents parameters, *Z* is the latent variable, *X* is the observed variable, *L* is the observed variable, *t* denotes the iteration number. This step selects the optimal output capsule by evaluating the response of each capsule to the observed data. In the M-step, our objective is to maximize the *Q* function. The M-step equation is as follows:

$$\theta^{t+1}=\arg\;\max_{\theta }Q(\theta |\theta^{t})$$ (2)

The *Q* function is defined as the expectation *θ*^*t*^ of the complete data log-likelihood function *L* of the observed data *X* with respect to *Z*, given the current parameter estimates. This expectation is used to update the model parameters in each iteration of the EM algorithm. The convergence property of the EM algorithm ensures that $\log L(\theta^{t}|X)$ does not decrease after each iteration, ultimately converging to a local maximum or saddle point.

To better understand the process, here is a specific example:

Assume we have a simple binary classification capsule network where each input vector can belong to two different capsule output classes. The initial parameter settings are *θ*^(0)^, which includes the means $\mu_{1}^{\left(0\right)}$ and $\mu_{2}^{\left(0\right)}$, and the variances $\sigma_{1}^{\left(0\right)}$ and $\sigma_{2}^{\left(0\right)}$ for the two classes. The E-step uses Bayes’ theorem to calculate the posterior probability *x*_*i*_ of each observed data point *γ* belonging to each class. In the *t*-th iteration, we compute the probability of each input vector *x*_*i*_ belonging to the two output capsules. Given the observed data *X* = {*x*_1_,*x*_2_,…,*x*_*N*_} and the latent variables *Z* = {*z*_1_,*z*_2_,…,*z*_*N*_}, the posterior probability is calculated using Bayes’ theorem as follows:

$$\gamma_{i1}^{\left(t\right)}=P(z_{i}=1|x_{i},\theta^{\left(t\right)})=\frac{\pi_{1}^{\left(t\right)}N(x_{i}|\mu_{1}^{\left(t\right)},\sigma_{1}^{\left(t\right)})}{\pi_{1}^{\left(t\right)}N(x_{i}|\mu_{1}^{\left(t\right)},\sigma_{1}^{\left(t\right)})+\pi_{2}^{\left(t\right)}N(x_{i}|\mu_{2}^{\left(t\right)},\sigma_{2}^{\left(t\right)})}$$ (3)

In this context, *N* represents a Gaussian distribution, *π* denotes the mixing coefficient, and $\gamma_{i1}^{\left(t\right)}$ indicates the probability that, at the *t*-th iteration, the *i*-th sample *x*_*i*_ belongs to the first output capsule. The M-step uses the posterior probabilities calculated in the E-step to update the parameters. The formulas for updating the mean and variance are as follows:

$$\mu_{1}^{\left(t+1\right)}=\frac{\sum_{i=1}^{N}\gamma_{i1}^{\left(t\right)}x_{i}}{\sum_{i=1}^{N}\gamma_{i1}^{\left(t\right)}}$$ (4)

$$\sigma_{1}^{\left(t+1\right)}=\frac{\sum_{i=1}^{N}\gamma_{i1}^{\left(t\right)}{\left(x_{i}-\mu_{1}^{\left(t+1\right)}\right)}^{2}}{\sum_{i=1}^{N}\gamma_{i1}^{\left(t\right)}}$$ (5)

By iteratively calculating the posterior probabilities and updating the parameters in this manner, the EM algorithm continuously optimizes the model parameters. Ultimately, it converges to a local maximum or saddle point, thereby selecting the optimal capsule path to enhance information flow.

## 3 Model topology

**Fig 1:**
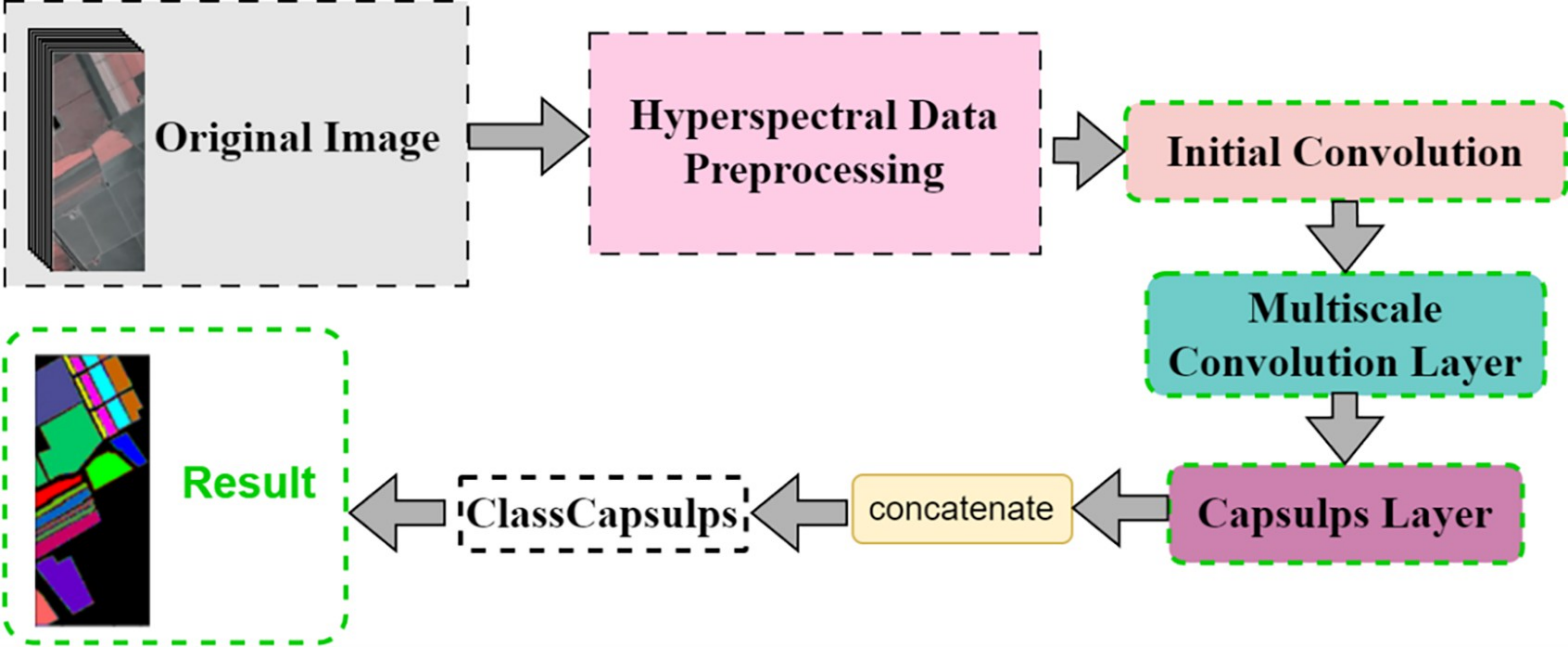
The topology of this model.

In this section, the design of the Multi-Scale Deep Separable Network (MDSC-Net) model is presented. Its overall structure is depicted in Fig 1. The architecture of the model is divided into six parts, each responsible for different processing and analytical tasks. The first part is dedicated to data preprocessing, where dimensionality reduction is conducted through Principal Component Analysis (PCA), followed by edge padding and image segmentation. In the second part, the initialization layer employs a 5×5 convolutional kernel to preliminarily extract features, providing a foundation for deep learning. The third part utilizes a multi-scale convolutional structure akin to the Inception module, extracting rich features through convolutional kernels ranging from 3×3 to 11×11, thereby enhancing the model’s ability to recognize details. The fourth part consists of a capsule layer, including a primary capsule layer and two intermediate al capsule layers. In the fifth part, an integration layer is employed, where the extracted multi-scale features are concatenated. Finally, the classification is performed in the classification capsule layer, where image classification is determined based on the activation values in the feature maps.

### 3.1 Data preprocessing module

**Fig 2:**
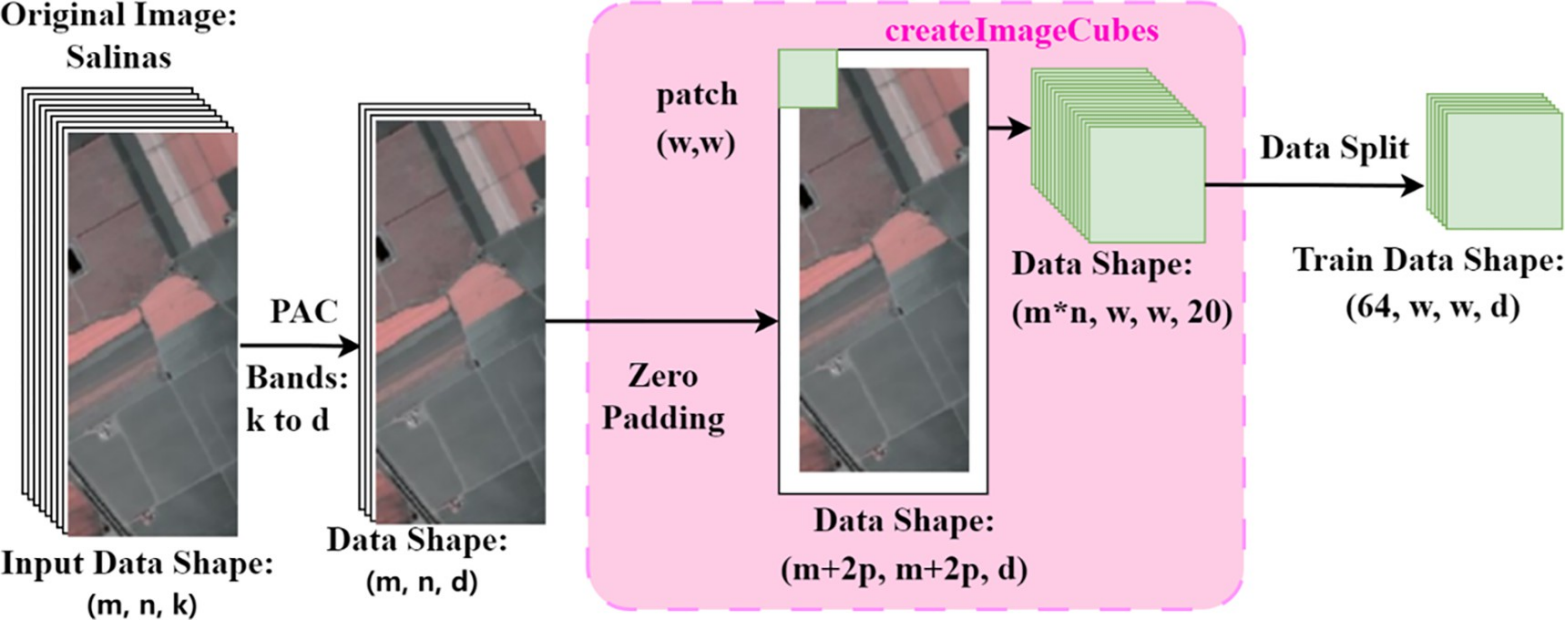
Data preprocessing.

The preprocessing stage of the data, as illustrated in Fig 2, aims to enhance computational efficiency, reduce data redundancy, and adjust the data to meet the input requirements of the model.

The original hyperspectral dataset possesses a spatial resolution of *m×n* and consists of *k* spectral bands. Initially, PCA is performed to reduce the dimensionality of the dataset, compressing it from *k* spectral bands to *d* bands that contain the most information. This not only improves computational efficiency and reduces data redundancy but also mitigates the impact of noise by discarding those bands that contain less information, as these bands are more susceptible to noise interference. Subsequently, to adapt to the model’s input requirements and preserve information at the edges, zero-padding is applied to the dataset. Specifically, considering a selected window size *w*, a zero boundary with a width of p pixels is added around all four sides of the dataset, altering its dimensions to (*m+2p*)×(*m+2p*)×*d*.

Next, a pixel-wise sliding window operation is performed on the padded image using *w×w* pixel blocks (patches), with a stride of 1, ensuring every original pixel can serve as the central point. This process generates *m×n* samples, reflecting the region-based approach of the model. Given the prevalence of non-informative background information in the dataset, which is not conducive to the training of the model, samples identified with a background label (label value 0) have been systematically eliminated.

In each training iteration, 64 samples are sequentially selected from the dataset to form a batch. Following the aforementioned preprocessing steps, the input data format for the model is finalized as 64×*w×w×d*.

### 3.2 Initial convolution and activation module

**Fig 3:**
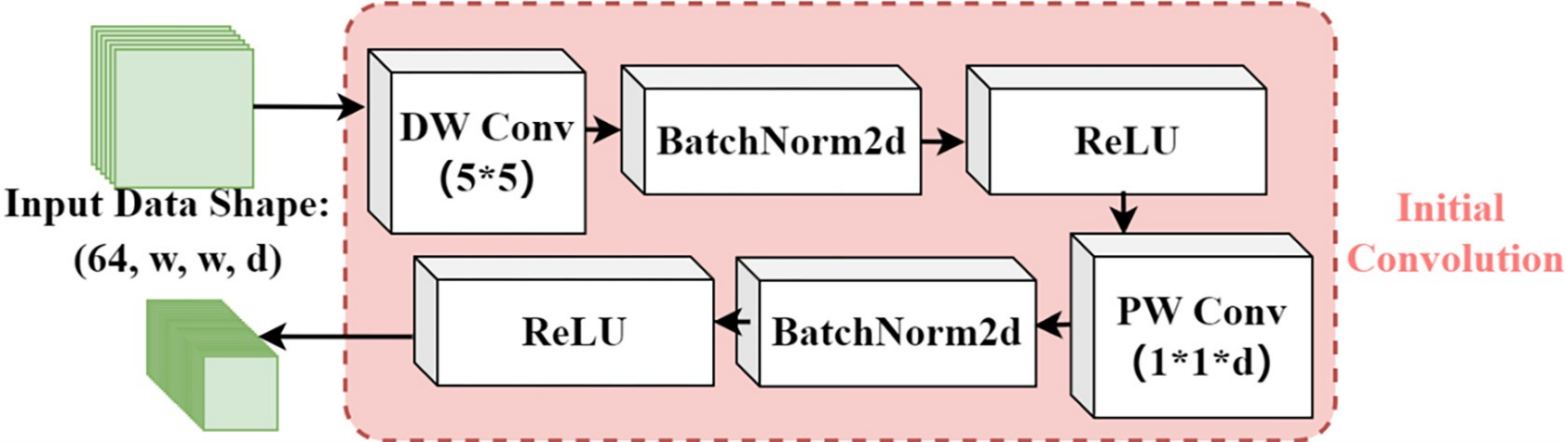
Initialization convolution module.

During the initialization phase of the model, as shown in Fig 3, the aim is to enhance the model’s ability to learn and extract complex features from the raw data. Specifically, the first deep convolutional layer (Deep Convolutional Convolution, DW Conv) employs d 5×5 convolutional kernels to extract spatial features from each independent channel.

Subsequently, the data processed by the convolutional layer are subjected to a normalization layer and a Rectified Linear Unit (ReLU) activation layer. The normalization layer standardizes the convolved data, enhancing the model’s generalization capability and accelerating the training process. It also aids in reducing noise interference by ensuring that all features are scaled equally, mitigating the unequal impact of noise on features at different scales. The ReLU layer introduces non-linearity, thereby boosting the model’s ability to learn complex data structures. After processing through these two layers, the structural dimensions of the data remain unchanged.

**Fig 4:**
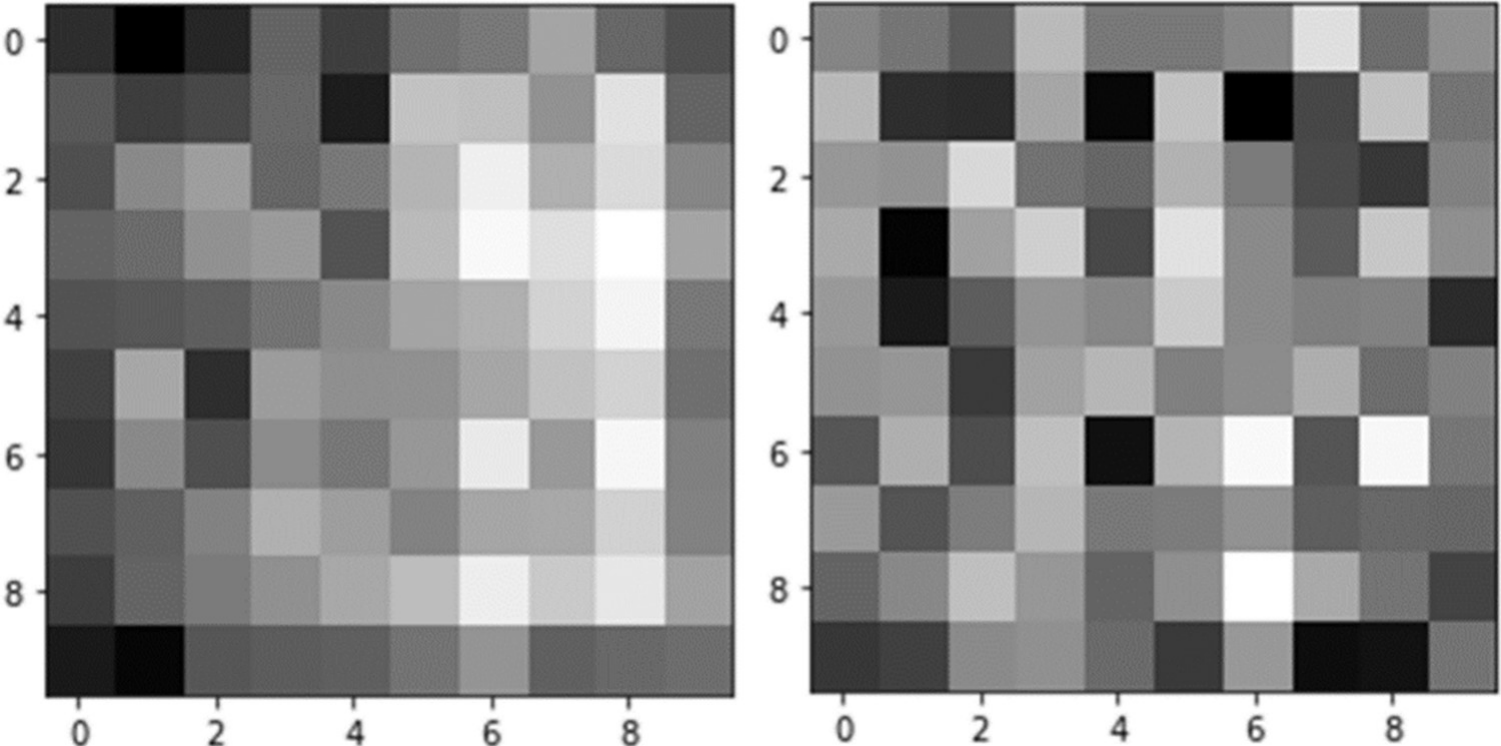
Initial convolution feature maps.

The pointwise convolution layer (PW Conv), using 64 1×1×*d* convolutional kernels, follows next. It integrates the outputs from the normalization and activation layers and adjusts the number of output channels. Another pass through a normalization layer and a ReLU activation layer completes the feature extraction function of the initialization convolution module on the raw data. In this manner, 64 distinct feature maps are extracted from the channels of the raw data, with two example feature maps illustrated in Fig 4.

### 3.3 Multi-scale capsule convolution module

**Fig 5:**
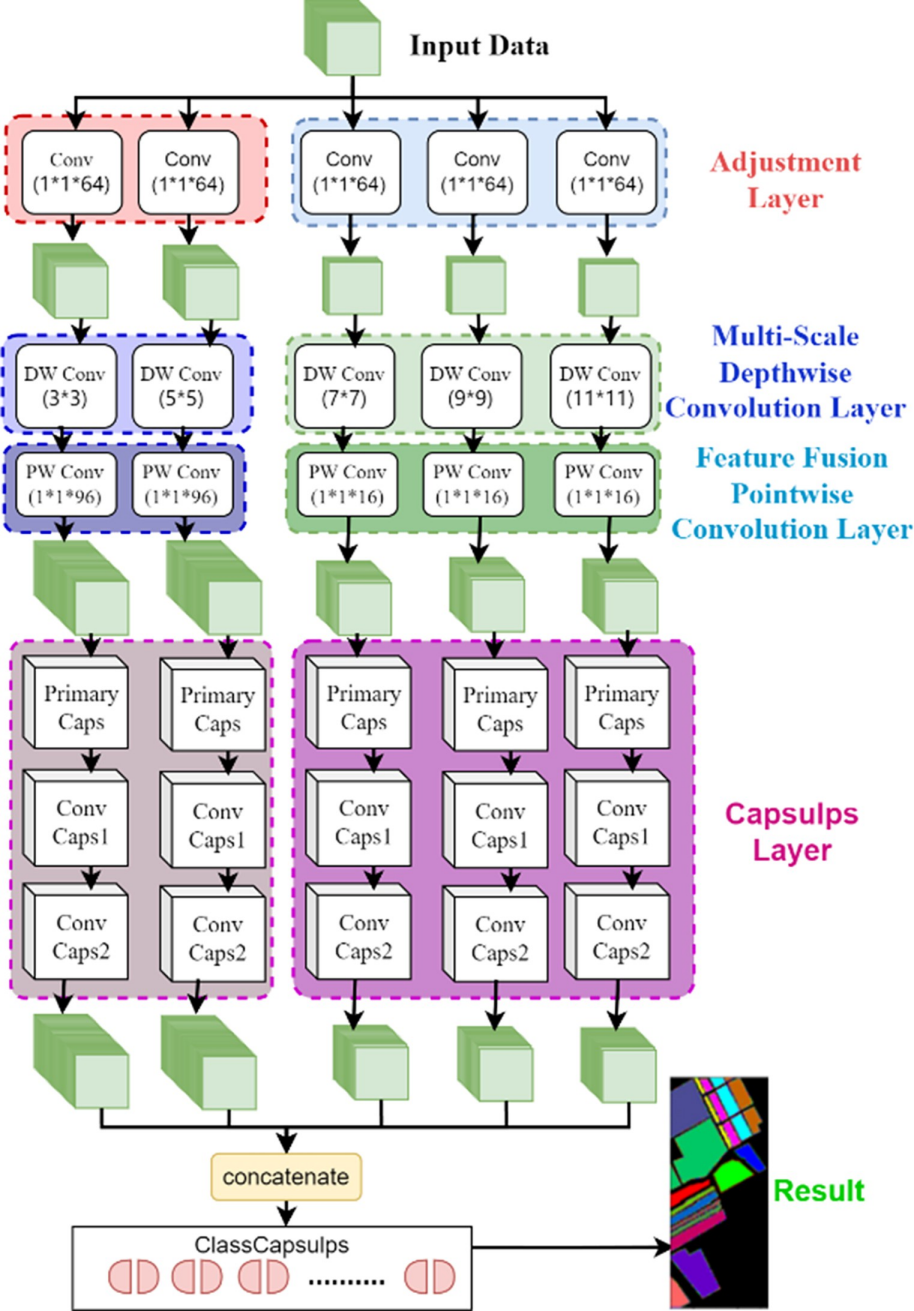
Multi-scale capsule convolution module.

The multi-scale capsule convolutional module, illustrated in Fig 5, is designed to extract multi-scale features and pose information from HSI, thereby enhancing the model’s performance and classification accuracy on complex datasets. The module employs a structure akin to the Inception model for processing data activated by ReLU. The initial stage includes an adjustment layer with 1×1×64 convolutional kernels, aimed at modulating the number of feature maps for subsequent multi-scale convolutional layers.

Specifically, for the paths processing 3×3 and 5×5 convolutional kernels, the output of this adjustment layer comprises 96 feature maps. The subsequent 3×3 and 5×5 depth convolutional layers further process these feature maps, ultimately outputting 128 feature maps through a 1×1×96 pointwise convolutional layer. This design is intended to capture more refined spatial features while enhancing the feature extraction capability by increasing the number of convolutional kernels.

On the other hand, for paths with larger convolutional kernels (i.e., 7×7, 9×9, 11×11), the adjustment layer outputs 16 feature maps. After processing by 7×7, 9×9, 11×11 depth convolutional layers and a 1×1×16 pointwise convolutional layer, each layer ultimately outputs 64 feature maps. This approach not only reduces the number of parameters but also effectively captures coarse spatial features by adjusting the use of convolutional kernels of different sizes, thereby achieving a balance between model simplification and precise feature extraction in HSI processing.

Following these convolutional layers is a primary capsule layer that integrates pose information and activation values. Subsequently, the model incorporates two intermediate capsule layers. Ultimately, the feature maps output by the five different convolutional kernel pathways are concatenated along the feature map dimension. At the end of the model, classification is performed by a classification capsule layer. Unlike traditional fully connected layers, this classification layer uses the activation values within the feature maps to vote, facilitating effective classification.

### 3.4 Capsule layer module

**Fig 6:**
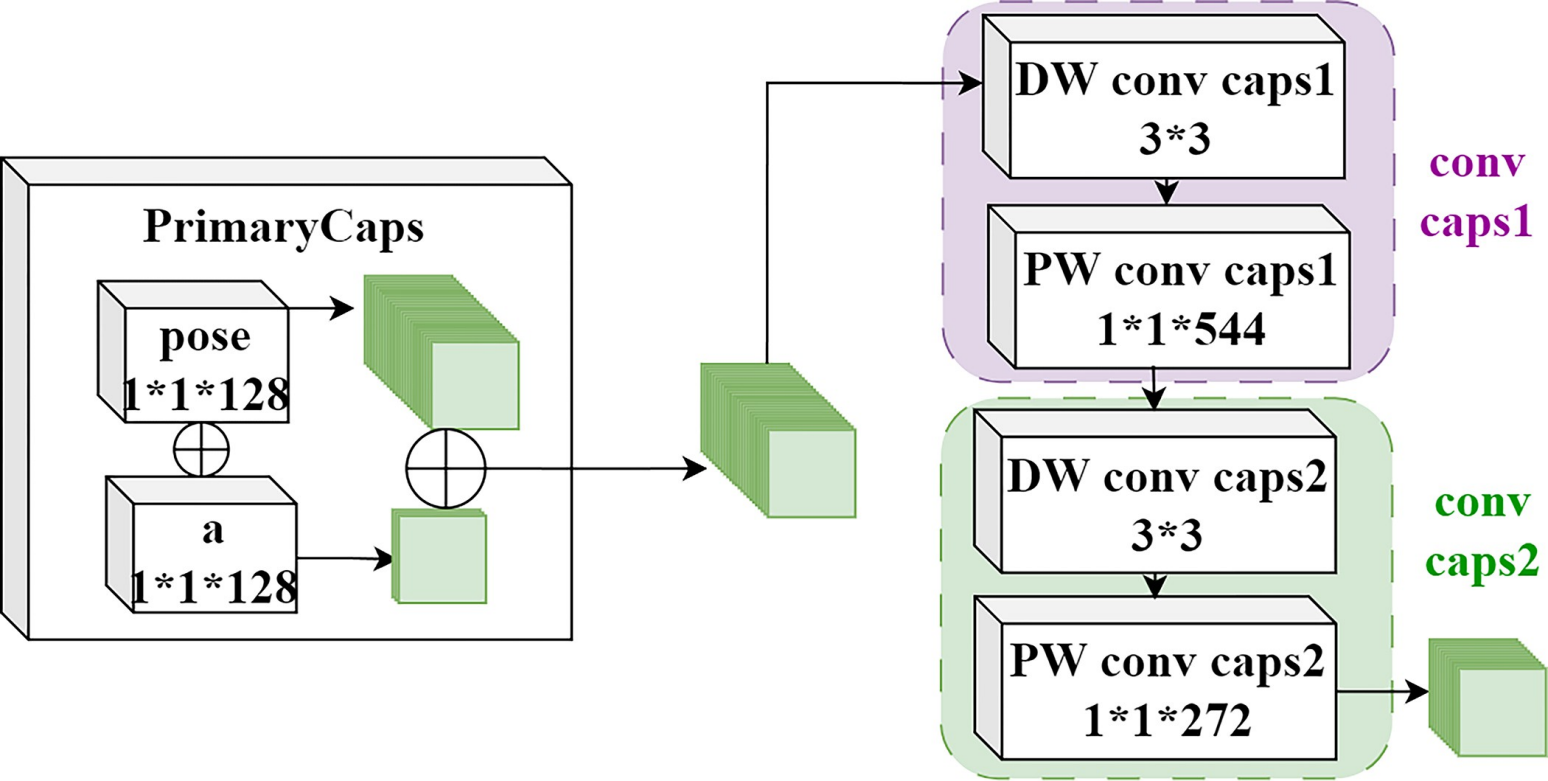
Capsule layer module.

The capsule layer structure within the multi-scale capsule convolution module is illustrated in Fig 6. The Primary Capsule Layer enhances the richness and precision of feature representation by integrating pose information and activation values. In this layer, the calculation formula for pose information is *B×P*^*2*^, where *B* represents the number of capsule types and *P* denotes the dimensions of the pose matrix. Using a 1×1×128 convolutional kernel, the pose layer generates *B×P* feature maps containing pose information. Similarly, the activation layer employs a 1×1×128 convolutional kernel to produce 32 feature maps containing activation values, culminating in a feature map dimension of *B×P*+32. Subsequently, the model incorporates two intermediate capsule layers, both utilizing the Expectation-Maximization (EM) routing algorithm for optimized processing. Specifically, the first intermediate capsule layer comprises a 3×3 depth convolutional layer and a 1×1×544 pointwise convolutional layer, aimed at capturing more complex feature information. The second intermediate capsule layer includes a 3×3 depth convolutional layer and a 1×1×272 pointwise convolutional layer, further refining feature extraction and processing.

### 3.5 Loss function

In capsule networks, the transformation matrix *W* converts the outputs of lower-level capsules (a group of neurons) into the inputs for higher-level capsules, allowing the lower-level capsules to predict the activation states of the higher-level capsules. The MDSC-Net employs a spread loss function for training this transformation matrix *W*. This loss function is specifically designed for optimizing capsule networks, aiming to diminish the impact of model weight initialization and to streamline the hyperparameter tuning process. The calculation of Li’s spread loss is as follows:

$$Li={\left(max(0,m-(at-ai))\right)}^{2}$$ (6)

Here, *m* represents the margin, while *at* and *ai* denote the activations of the target class *t* and class *i*, respectively. The model incurs a penalty for the squared difference between predictions and the margin *m* when the disparity between the true class and other classes is less than *m*. To prevent the emergence of ineffective capsules at the onset of training, the initial margin *m* is set to a lower value of 0.2.

### 3.6 Model architecture

**Fig 7:**
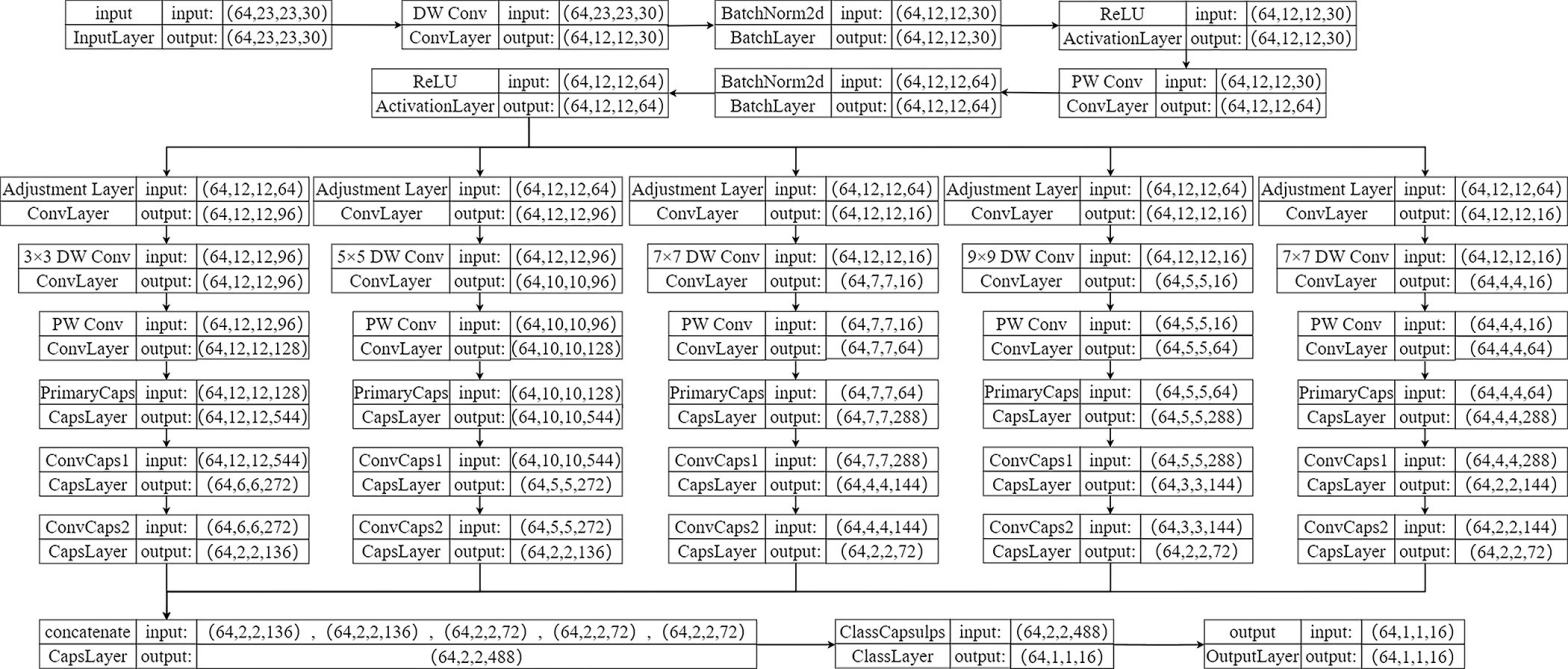
The proposed MDSC-Net architecture. The final layer of this architecture is specifically designed using the Salinas dataset.

The detailed explanation of the MDSC-Net attention-dense network model includes the types of layers used, the dimensions of the generated output maps, and the required number of parameters. The specific layer types and their corresponding parameters are illustrated in detail in Fig 7.

## 4 Experimental results and analysis

### 4.1 Experimental setup and dataset description

All the expriments and testing environment were carried out on the PyTorch 1.10.2 framework, accelerated with CUDA 11.3. The Adam optimizer was selected to combine the exponential decay learning rate to optimiza the strategy training model. The initial learning rate was set at 0.003 and decreased with a decay rate of 0.96 as the number of iterations increased. The batch size is 64 for all cases. To achieve optimal model performance for each dataset, epochs are 400 for KSC Dataset, 300 for Pavia University and 200 for Salinas Dataset. The training was performed in a desktop computer running Windows 11, an Intel Core i7-12700H processor, an NVIDIA RTX 3070 Ti graphics card with 8GB of VRAM, and 16GB of system RAM.

To comprehensively evaluate the performance of the newly proposed MDSC-Net model, three available hyperspectral remote sensing imaging datasets were selected: the Kennedy Space Center Dataset, the Pavia University Dataset, and the Salinas Dataset. The details of the dataset are as follows:

1. The KSC Dataset was acquired from the area around the Kennedy Space Center, USA. It is made up of 13 categories across 224 spectral bands, reduced to 176 after removing water vapor noise. The resolution of the original image is 512×614 pixels, equating to an 18m×18m area per pixel. For computational efficiency in the MDSC-Net model, the band count was reduced to 20 through PCA and the images were resized to 532×634 pixels, further segmented into 21×21 pixel blocks. All background pixel blocks (labeled as zero) were excluded from the experimental dataset. The category details of the Kennedy Space Center dataset are shown in Table 1. In the entire dataset, there are 260 samples in the training set, 1823 samples in the validation set, and 3128 samples in the test set, with the training set comprising 5% of the total samples.

**Table 1:** Category details of the KSC dataset.

Index	Land Cover Type	Training Set Samples	Validation Set Samples	Test Set Samples	Total Samples
**0**	Scrub	38	266	457	761
**1**	Willow swamp	12	85	146	243
**2**	CP hammock	13	90	153	256
**3**	Slash pine	13	88	151	252
**4**	Oak/broadleaf	8	56	97	161
**5**	Hardwood	11	80	138	229
**6**	Swamp	5	37	63	105
**7**	Graminoid marsh	22	151	258	431
**8**	Spartina marsh	26	182	312	520
**9**	Cattail marsh	20	141	243	404
**10**	Salt marsh	21	147	251	419
**11**	Mud flats	25	176	302	503
**12**	Water	46	324	557	927

2. The Pavia University Dataset was collected by the University of Pavia, Italy, which is widely used in hyperspectral image classification and clustering algorithm research. The dataset is 610×340 pixels in size, has a spatial resolution of 1.3 meters, and initially contains 103 bands. It covers three different scenes: urban, rural, and wilderness, showcasing a variety of ground objects, including various buildings, land covers, and plant types. After compressing to 20 bands and dividing into 19×19 pixel blocks, the category details of the Pavia University dataset are shown in Table 2. In the entire dataset, there are 4277 samples in the training set, 11549 samples in the validation set, and 26950 samples in the test set, with the training set comprising 10% of the total samples.

**Table 2:** Category details of the Pavia University dataset.

Index	Land Cover Type	Training Set Samples	Validation Set Samples	Test Set Samples	Total Samples
**0**	Asphalt	663	1989	3979	6631
**1**	Meadow	1865	5595	11189	18649
**2**	Metal sheets	135	406	812	1354
**3**	Shadows	95	284	568	947
**4**	Gravel	210	630	1259	2099
**5**	Bitumen	133	399	798	1330
**6**	Bricks	368	1105	2209	3682
**7**	Bare soil	503	1509	3017	5029
**8**	Trees	306	919	1839	3064

3. Designed for hyperspectral image classification, the Salinas Dataset was obtained around the region of California’s Salinas Valley, USA, by using the Airborne Visible/Infrared Imaging Spectrometer (AVIRIS). It features images at 512×217 pixels, a 3.7m spatial resolution, and originally 224 bands, reduced to 204 after noise removal. The ground truth consists of 16 types of features, including vegetables, bare soil, and vineyards. The original image is preprocessed to obtain blocks with 30 bands and a size of 23×23 pixels. The category details of the Salinas dataset are also shown in Table 3. In the entire dataset, there are 2706 samples in the training set, 25711 samples in the validation set, and 25712 samples in the test set, with the training set comprising 5% of the total samples.

**Table 3:** Category details of the Salinas dataset.

Index	Land Cover Type	Training Set Samples	Validation Set Samples	Test Set Samples	Total Samples
**0**	Brocoligreenweeds_1	101	704	1204	2009
**1**	Brocoligreenweeds_2	186	1304	2236	3726
**2**	Fallow	99	692	1185	1976
**3**	Fallowroughplow	70	488	836	1394
**4**	Fallow_smooth	134	937	1607	2678
**5**	Stubble	198	1386	2375	3959
**6**	Celery	179	1253	2147	3579
**7**	Grapes_untrained	564	3945	6762	11271
**8**	Soilvinyarddevelop	310	2171	3722	6203
**9**	Cornsenescedgreen_weeds	164	1147	1967	3278
**10**	Lettuceromaine4wk	53	374	641	1068
**11**	Lettuceromaine5wk	96	674	1157	1927
**12**	Lettuceromaine6wk	46	321	549	916
**13**	Lettuceromaine7wk	54	375	641	1070
**14**	Vinyard_untrained	363	2544	4361	7268
**15**	Vinyardverticaltrellis	90	632	1085	1807

### 4.2 Evaluation indicators

Different widely utilized quantitative measurement methods are utilized for assessing the network model proposed. These include Overall Accuracy (OA), which reflects the proportion of correctly classified samples, serving as an intuitive performance measure; Average Accuracy (AA), ensuring balanced performance assessment across different categories; Precision, measuring the accuracy of predicted positive samples; Recall, indicating the proportion of actual positives correctly predicted; the Kappa coefficient, evaluating the model’s performance surpassing random levels; and the F1 Score (F1), providing a comprehensive assessment of classification accuracy and recall by calculating the harmonic mean of Precision and Recall. The formulae for these metrics are as follows:

$$OA=\frac{1}{N}\sum_{i=1}^{r}x_{ii}$$ (7)

$$AA=\frac{1}{N}\sum_{i=1}^{r}Accuracy_{i}$$ (8)

$$\mathrm{Pr}ecision=\frac{TP}{TP+FP}$$ (9)

Recall=TPTP+FN (10)

$$Kappa=\frac{N\sum_{i=1}^{r}x_{ii}-\sum_{i=1}^{r}\left(Nx_{i.}\right)}{N^{2}-\sum_{i=1}^{r}\left(Nx_{i.}\right)}$$ (11)

$$F_{1}=\frac{2*Precision*Recall}{Recall+Precision}$$ (12)

In the context of the equation, is the total number of categories; *N* is the total number of samples; *x*_*ii*_ represents the number of samples of class *i* correctly identified as class *i*; *Accuracy*_*i*_ is the precision of class *i*; True Positives (*TP*) denotes the number of actual positives correctly identified; False Positives (*FP*) indicates incorrect positive identifications among negatives; False Negatives (*FN*) represents missed positives, where actual positives are incorrectly classified as negatives.

### 4.3 Results of ablation studies

To validate the effectiveness of the proposed MDSC-Net model, ablation experiment were conducted on the Salinas dataset. The performance of different models was compared using three metrics: Overall Accuracy (OA), Average Accuracy (AA), and Kappa Score(Kappa), with consistent parameter settings and training strategies across models. Two comparative models have been selected: one is the capsule network model utilizing single-scale instead of multi-scale convolution kernel, and the other is a multi-scale convolutional network model using max-pooling layer directly instead of dynamic routing mechanism in the capsule network. Description of each model is shown in Table 4, with the comparative results of the ablation studies presented in Table 5.

**Table 4:** Combination structure of MDSC-Net.

Network Model	Description
**MDSC-0**	Capsule network model utilizing only single-scale convolutional kernels
**MDSC-1**	Multi-scale convolutional network model employing max-pooling layers
**MDSC-Net**	Multi-scale network model with an alternative to max-pooling layers

**Table 5:** Results of ablation experiment.

Network Model	OA(%)	AA(%)	Kappa(%)
**MDSC-0**	97.42	97.01	98.02
**MDSC-1**	92.43	91.96	92.36
**MDSC-Net**	99.31	98.86	99.23

Table 5 shows that the overall performance of MDSC-Net has been significantly improved after extracting multi-level features through multi-scale convolution kernel and replacing the max-pooling layer with the dynamic routing mechanism in capsule network. Specifically, compared with MDSC-0 (the capsule network model with single-scale convolutional kernel), MDSC-Net has improved the three key performance indexes of OA, AA and Kappa by 1.94%, 1.91% and 1.23% respectively. Against MDSC-1 (the multi-scale convolutional network model with max-pooling layer), the improvements were 7.44%, 7.50%, and 7.44% in the same metrics. The comparison between MDSC-0 and MDSC-Net highlights that the multi-scale convolutional kernel can capture rich multi-level features, thus effectively enhancing the capsule network’s capability to process ground detail and multi-level information in HSI. The comparison between MDSC-1 and MDSC-Net indicates that substituting traditional max-pooling with the dynamic routing mechanism of capsule networks prevents the loss of precise location and posture information of objects, and realizes the translation invariance of the enhanced model features, and further improve the ability of feature extraction.

**Fig 8:**
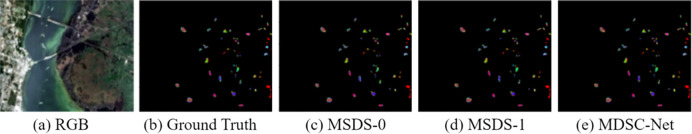
Figure of intermediate results of ablation experiment.

The classification results of ablation experiments are presented in Fig 8, including: (a) the original image of the dataset; (b) the manually annotated ground truth; (c) the output from the capsule network model using only single-scale convolutional kernel; (d) the output from the multi-scale convolutional network model with max-pooling layer; and (e) the output from the MDSC-Net model, which displayed more refined features compared to (c) and (d). This result is attributed to MDSC-Net’s ability to extract rich hierarchical features through the application of multi-scale convolutional kernel, and effectively preserves object posture information via the capsule network, thereby more comprehensively extracting the feature details of HSI.

### 4.4 Results of comparative studies

To verify the effectiveness of the proposed MDSC-Net model, comparisons were made with existing models on the Kennedy Space Center dataset, the Pavia University dataset, and the Salinas dataset. The comparative models included SPP [28], DCNN [29], 3-D CNN [30], SPL-SR [31], AFLA-SCNN [32], and MLGSC [33]. Quantitative analysis was performed using the metrics Overall Accuracy (OA), Average Accuracy (AA), Recall, and F1 Score(F1).

**Fig 9:**
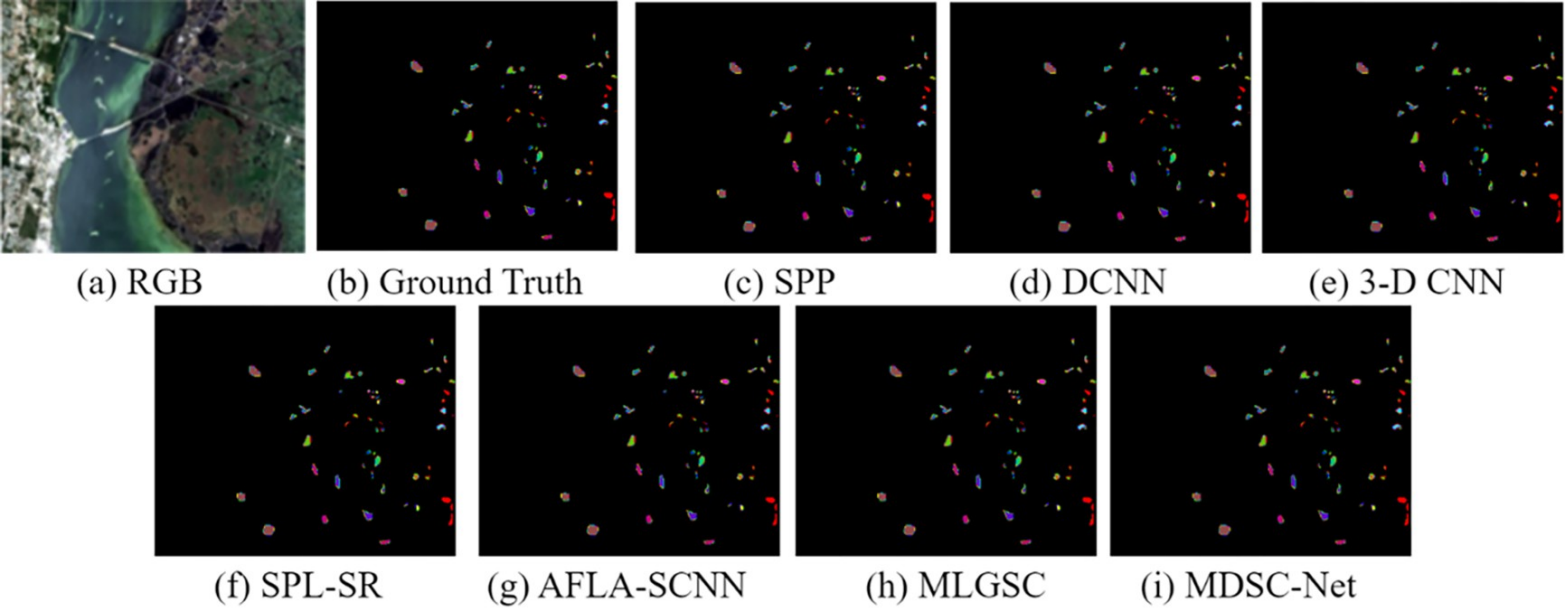
Qualitative comparative experimental results on the KSC dataset.

The first comparative experiment, as shown in Fig 9, demonstrates the classification results of various classification models on the Kennedy Space Center dataset. Different subgraphs are labeled in the Figure: (a) the RGB composite image; (b) the manually annotated ground truth; and (c) to (h) correspond to the outputs of several advanced classification techniques, including SPP, DCNN, 3-D CNN, SPL-SR, CNN_HSI, SpectralNET, and the proposed MDSC-Net model in (i).

Table 6 shows the quantitative analysis of the segmentation results on Kennedy Space Center dataset. The MDSC-Net model shows better classification accuracy than all other methods with AA, OA, FI, and Recall of 93%, 94%, 94%, and 92% respectively. The experiment shows that compared with SPP, DCNN, and 3-D CNN, the comparative metrics AA, OA, Recall, and F1 Score improved by 1% to 8% respectively. The overall parameter is higher than CNN_HSI and lower than SpectralNET. It can be concluded that the proposed MDSC-Net provides superior classification results on the Kennedy Space Center dataset with an acceptable parameter volume.

**Table 6:** Quantitative comparative experimental results on the KSC dataset.

Quantitative Metrics	SPP	DCNN	3-D CNN	SPL-SR	AFLA-SCNN	MLGSC	MDSC-Net
**AA(%)**	92 ± 0.1	92±0.3	86±0.4	91±0.3	91±0.02	88±0.01	**92±0.1**
**OA(%)**	91 ± 0.1	93±0.3	93±0.3	92±0.3	92±0.01	89±0.02	**94±0.2**
**F1(%)**	95 ± 0.1	94±0.03	86±0.4	92±0.3	92±0.03	89±0.02	**93±0.3**
**Recall(%)**	93 ± 0.1	0.93±0.3	93±0.3	93±0.3	91±0.01	87±0.07	**94±0.2**

**Fig 10:**
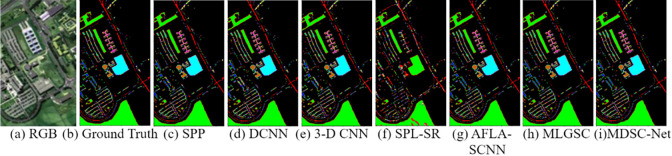
Qualitative comparative experimental results on the Pavia University dataset.

The second experiment, illustrated in Fig 10, presents the classification results of various models on the Pavia University dataset, with our proposed method achieving the most accurate segmentation results.

Table 7 lists the segmentation results on the Pavia University dataset. Compared to the six comparative models, the MDSC-Net has shown significant improvements in AA, OA, FI, and Recall by 97%, 98%, 94%, and 92% respectively. In summary, the MDCS-Net exhibited better segmentation outcomes than the benchmark algorithms on the Pavia University dataset. It showed superior performance with reasonable computational parameters, offering significant advantages over other models. These results confirm the robustness and broad applicability of MDSC-Net.

**Table 7:** Quantitative comparative experimental results on the Pavia University dataset.

Quantitative Metrics	SPP	DCNN	3-D CNN	SPL-SR	AFLA-SCNN	MLGSC	MDSC-Net
**AA(%)**	95 ± 0.3	88±0.4	94±0.3	88±0.4	94±0.03	94±0.04	**97±0.2**
**OA(%)**	93 ± 0.4	92±0.3	94±0.3	84±0.4	93±0.03	95±0.03	**98±0.1**
**F1(%)**	94± 0.3	89±0.2	92±0.1	86±0.2	95±0.03	94±0.02	**98±0.1**
**Recall(%)**	96 ± 0.2	90±0.3	94±0.3	89±0.4	95±0.03	95±0.03	**97±0.2**

**Fig 11:**
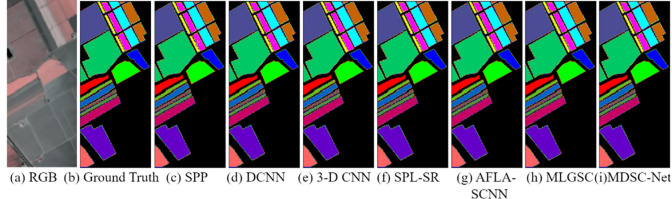
Qualitative comparative experimental results on the Salinas dataset.

In the third experiment, as shown in Fig 11, the proposed method achieved better classification results on the Salinas dataset, with finer extraction results from minor to major categories, closely approximating the manually annotated Ground Truth.

As can be seen from Table 8, for Salinas dataset, the proposed method still maintained the most advanced performance, with AA, OA, F1 Score, and Recall all at 99%.

**Table 8:** Quantitative comparative experimental results on the Salinas dataset.

Quantitative Metrics	SPP	DCNN	3-D CNN	SPL-SR	AFLA-SCNN	MLGSC	MDSC-Net
**AA(%)**	81±0.3	93±0.3	98±0.2	96±0.2	98±0.02	97±0.03	**99±0.1**
**OA(%)**	76±0.4	89±0.3	96±0.2	93±0.2	97±0.02	98±0.03	**99±0.4**
**F1(%)**	83±0.2	92±0.5	97±0.3	95±0.2	98±0.02	97±0.02	**99±0.2**
**Recall(%)**	78±0.3	87±0.3	99±0.2	94±0.3	97±0.03	99±0.04	**99±0.1**

For hyperspectral remote sensing image classification, some models are complex [34–36], while others capture insufficient semantic information [37–39], necessitating efficient classification algorithms that ensure high accuracy while quickly processing large volumes of remote sensing image data. The introduction of multi-scale convolution strategy increases the computational complexity due to the parallel computation involving multiple scale convolution layers. To address this, the deep separable convolution technique is used to independently process the spatial feature extraction of each channel through two-dimensional convolution kernel, thereby reducing the number of parameters without sacrificing the overall model accuracy.

**Fig 12:**
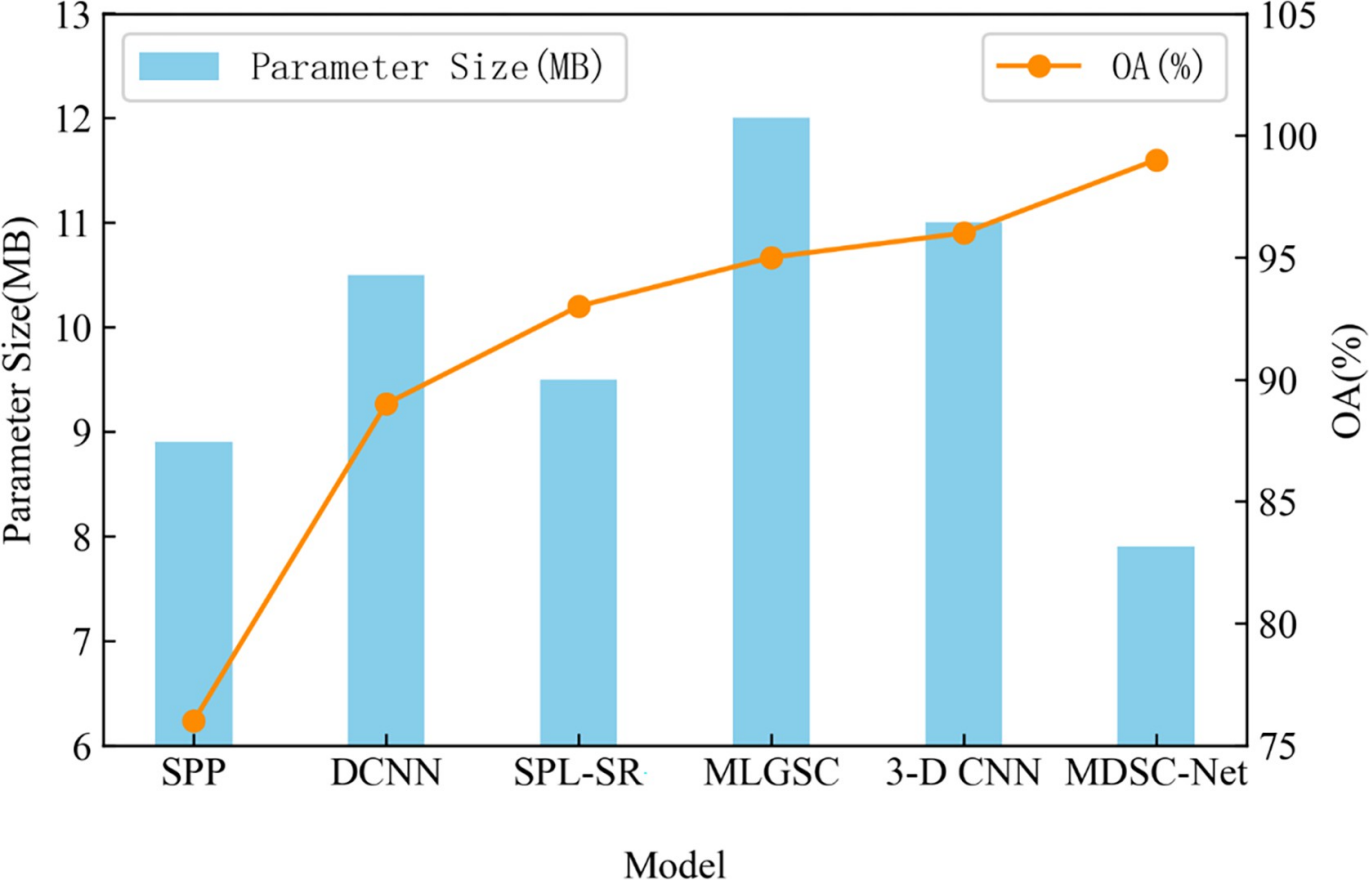
Comparison chart of model parameter quantity and OA.

Traditional convolution operations handling spatial feature extraction and channel fusion as one unit lead to a significant parameter count (i.e., 64×k×k×d), while depthwise separable convolution partitions these processes. This separation significantly reduces the total number of parameters. Spatial feature extraction is conducted with parameters of k×k×d, while channel fusion is done with parameters of 64×1×1×d. As depicted in Fig 12 (parameter volume represented by blue bars and average accuracy by red lines), the proposed MDSC-Net demonstrates superior accuracy compared to the hierarchical multi-scale concatenation net (HMC-Net) by effectively utilizing depthwise separable convolution.

This approach not only enhances accuracy but also reduces the parameter count significantly, making it more suitable for real-world image classification tasks. This is attributed to MDSC-Net’s adoption of multi-scale convolution kernels, depthwise separable convolutions, and capsule networks, which together enhance its ability to efficiently process and interpret complex image data while minimizing computational overhead.

### 4.5 Cross-validation experiment and analysis

**Fig 13:**
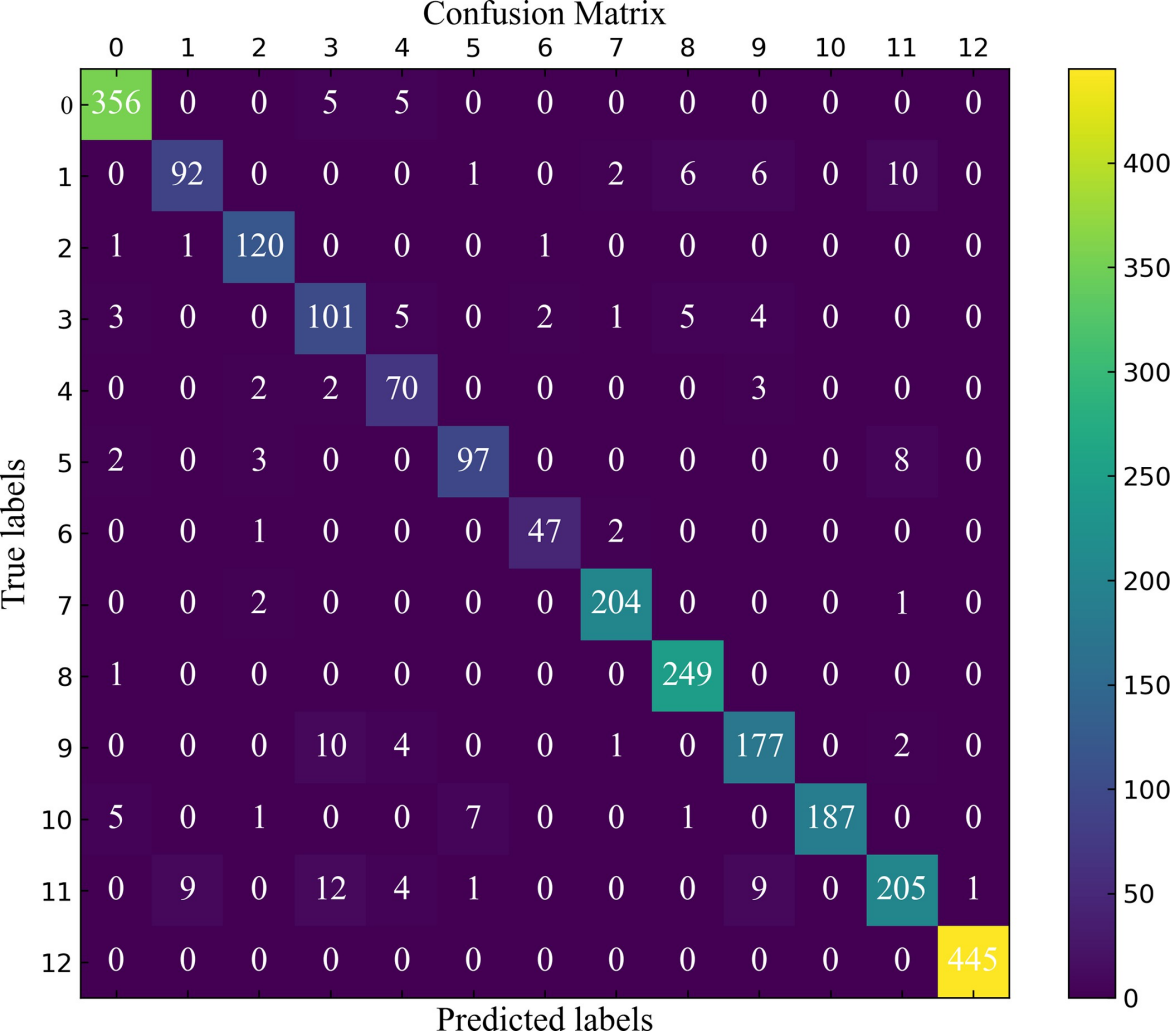
Confusion matrix.

To ensure the effectiveness and generalization ability of the proposed MDSC-Net model, this study employs 5-fold cross-validation on the Kennedy Space Center dataset. In the 5-fold cross-validation, the dataset is equally divided into five subsets, with each subset used as the test set in turn, while the remaining four subsets serve as the training set. This validation method effectively reduces random errors and the risk of overfitting during the model evaluation process, ensuring the reliability and consistency of the experimental results. The confusion matrix, averaged over the five test sets, is shown in the Fig 13.

From the comparative experiments, model parameter experiments, and cross-validation experiments, the following observations can be made:

By employing multi-scale convolutional kernels, depthwise separable convolutions, and capsule networks, MDSC-Net can effectively extract complex ground object information from hyperspectral images, maintain the translational invariance of features, and significantly reduce computational complexity and the number of parameters, thereby optimizing performance and efficiency.

Compared to state-of-the-art models, MDSC-Net performs better in terms of Overall Accuracy (OA), verifying its consistency and stability across all categories. Observing the confusion matrix, it is evident that MDSC-Net achieves high classification accuracy even in small sample categories. For instance, in categories 1, 4, 5, and 6 of the Kennedy Space Center dataset, despite having fewer training samples, the classification accuracy does not significantly decrease. However, the MDSC-Net model has certain limitations. Due to the reduction in the number of parameters, the time complexity is relatively increased, requiring approximately 4 hours of training on a computer equipped with a 3070 Ti GPU.

## 5 Conclusion

This article presents a Multi-Scale Depthwise Separable Capsule Network for hyperspectral image (HSI) classification, demonstrating significant effectiveness in the classification of hyperspectral imagery. By employing multi-scale convolutional kernels, the network is capable of capturing features across different scales, effectively deciphering the complex terrain details and hierarchical information in HSIs. Moreover, the model employs depthwise separable convolutions to streamline processing, achieving efficient feature extraction with reduced computational load. The incorporation of capsule networks, as an improvement over traditional max-pooling layers, allows the model to reduce the size of feature maps while maintaining translational invariance and preventing the loss of terrain posture information. Ablation studies show that the MDSC-Net, with its multi-scale convolutional kernels and capsule networks, significantly enhances HSI classification performance. Comparative experiments on three HSI datasets further affirm the performance advantages of this model over existing ones, including structural simplicity, lower computational complexity, and higher classification accuracy.

For future work, this model can be utilized in precision agriculture for crop health monitoring. By analyzing hyperspectral images, issues such as pest infestations and nutrient deficiencies can be identified, providing precise field management recommendations. Additionally, this model can have significant applications in urban planning. It can be used for land use classification and change detection, assisting planners in accurately assessing urban expansion and the dynamic changes in land use.

This study has some limitations, such as the potential for improved classification accuracy on datasets with a smaller number of samples. Additionally, the generalizability of the model to other hyperspectral image datasets remains to be verified. Challenges also exist regarding processing speed in real-time applications and computational demands on resource-limited devices like embedded systems. Future research should focus on exploring effective ways to handle large datasets under constrained computational resources. Incremental Principal Component Analysis (IPCA) and incremental learning methods can be employed to gradually extract the main components of the data, reducing the demand for computational resources and storage, and supporting dynamic model updates to lower computational costs. Moreover, the MDSC-Net model faces the challenge of maintaining efficient performance on battery-powered devices. This requires adjustments through quantization and sparsity techniques to reduce energy consumption and the exploration of adaptive computing methods to achieve an optimal balance between performance and energy consumption. Furthermore, investigating cross-modal learning strategies, such as combining HSI data with LiDAR data, may enhance the accuracy and robustness of HSI classification, thereby improving the performance and applicability of the MDSC-Net model in real-world scenarios. Finally, to enhance the model’s generalizability across different datasets, it is crucial to test and optimize it on a more diverse range of datasets.

## Supporting information

S1 File (ZIP)

## References

[1] Li S, Song W, Fang Y, Chen Y, Ghamisi P, et al. Deep learning for hyperspectral image classification: An overview. IEEE Transactions on Geoscience and Remote Sensing. 2023;59(1):1–18.

[2] Zhang Y, Wang J, Zhang J, et al. A survey on hyperspectral image processing techniques for environmental monitoring. Remote Sensing of Environment. 2023;258(1):1–19.

[3] Liu X, Liang Y, Wang Z. Hyperspectral remote sensing for land cover change detection: A review and meta-analysis. Remote Sensing of Environment. 2023;258(1):20–38.

[4] Landgrebe D. Hyperspectral image data analysis. IEEE Signal Processing Magazine. 2002;19(1):17–28. doi: 10.1109/79.974718

[5] Qu S, Li X, Gan Z, et al. A new hyperspectral image classification method based on spatial-spectral features. Remote Sensing. 2022;12(1):1–16. doi: 10.1038/s41598-022-05422-5 35087142 PMC8795209

[6] Fukushima K. Neocognitron: A self-organizing neural network model for a mechanism of pattern recognition unaffected by shift in position. Biological Cybernetics. 1980;36(4):193–202. doi: 10.1007/BF00344251 7370364

[7] Krizhevsky A, Sutskever I, Hinton GE, et al. ImageNet classification with deep convolutional neural networks. Communications of the ACM. 2017;60(6):84–90. doi: 10.1145/3065386

[8] He K, Zhang X, Ren S, et al. Deep residual learning for image recognition. 2016 IEEE Conference on Computer Vision and Pattern Recognition. IEEE, 2016:770–778.

[9] Szegedy C, Liu W, Jia Y, et al. Going deeper with convolutions. 2015 IEEE Conference on Computer Vision and Pattern Recognition. IEEE, 2015:1–9.

[10] Zhao R, Zhang C, Xue D. A multi-scale multi-channel CNN introducing a channel-spatial attention mechanism hyperspectral remote sensing image classification method. Eur J Remote Sens. 2024;57(1):1–14. doi: 10.1080/22797254.2024.2353290

[11] Tejasree G, Agilandeeswari L. An extensive review of hyperspectral image classification and prediction: techniques and challenges. Multimed Tools Appl. 2024;110(42):18562–18592.

[12] Yang JX, Zhou J, Wang J, Tian H, Liew AWC. HSIMamba: Hyperspectral Imaging Efficient Feature Learning with Bidirectional State Space for Classification. Remote Sens. 2024;16:1–12.

[13] Zhang Y, Li J, Zhang L, et al. Hyperspectral Image Classification Method Based on 2D–3D CNN and Multibranch Neural Network. IEEE Geoscience and Remote Sensing Letters. 2020;17(12):2106–10. doi: 10.1109/LGRS.2020.3006761

[14] Li Z, Huang W, Wang L, et al. A novel convolutional neural network for hyperspectral image classification. IEEE Transactions on Geoscience and Remote Sensing. 2021;11(1):1–16.

[15] Roy SK, Krishna G, Dubey SR, et al. HybridSN: Exploring 3-D–2-D CNN Feature Hierarchy for Hyperspectral Image Classification. IEEE Geoscience and Remote Sensing Letters. 2020;17(2):277–81. doi: 10.1109/LGRS.2019.2918719

[16] Chakraborty T, Trehan U. SpectralNET: Exploring spatial-spectral waveletCNN for hyperspectral image classification. 2021. doi: 10.48550/arXiv.2104.00341

[17] Wei L, Ma H, Yin Y, et al. Kmeans-CM Algorithm With Spectral Angle Mapper for Hyperspectral Image Classification. IEEE Access. 2023;11(1):26566–76.

[18] Yin Y, Wei L. Hyperspectral image classification using ensemble extreme learning machine based on fuzzy entropy weights and auto-adapted spatial-spectral features. In: Multimedia Tools and Applications. Springer; 2023:217–38.

[19] Hinton GE, Krizhevsky A, Wang SD. Transforming auto-encoders. In: Artificial Neural Networks and Machine Learning–ICANN 2011. Springer; 2011:4451–8.

[20] Sabour S, Frosst N, Hinton GE. Dynamic routing between capsules. In: Advances in Neural Information Processing Systems 30. Curran Associates, Inc.; 2017:3859–69.

[21] Zhang H, Meng L, Wei X, et al. 1D-Convolutional Capsule Network for Hyperspectral Image Classification. In: Artificial Neural Networks and Machine Learning–ICANN 2019. Springer; 2019:4451–8.

[22] Hinton GE, Sabour S, Frosst N. Matrix capsules with EM routing. In: International Conference on Learning Representations. OpenReview; 2018.

[23] Yang JX, Zhou J, Wang J, Tian H, Liew AWC. HSIMamba: Hyperpsectral Imaging Efficient Feature Learning with Bidirectional State Space for Classification. arXiv. 2024. doi: 10.48550/arXiv.2404.00272

[24] Wang W, Xu Y, Xu Z, Kong C, Niu X, Huang J. Multiscale Spectral–Spatial Capsule Neural Network for Hyperspectral Image Classification. In: Proceedings of the UNIfied Conference of DAMAS, IncoME and TEPEN Conferences (UNIfied 2023). 2024;151(1):185–194.

[25] Zhai H, Zhao J. Two-Stream Spectral-Spatial Convolutional Capsule Network for Hyperspectral Image Classification. Int J Appl Earth Obs Geoinf. 2024.

[26] Moraga YJ, Duzgun HS. JigsawHSI: a network for Hyperspectral Image classification. arXiv preprint arXiv:2206.02327v2, 2022. doi: 10.48550/arXiv.2206.02327

[27] Picco ML, Ruiz MS. Hyperspectral Image Classification Using Deep Matrix Capsules. IEEE Transactions on Geoscience and Remote Sensing, 2022. doi: 10.1109/TGRS.2022.3177196

[28] Li W, Du Q, Zhang H. A novel deep learning framework for hyperspectral image classification using spatial pyramid pooling. IEEE Transactions on Geoscience and Remote Sensing. 2023;61(3):1199–211.

[29] Zhang Y, Du B. Deep model based transfer and multi-task learning for hyperspectral image classification. IEEE Transactions on Geoscience and Remote Sensing. 2023;61(8):4759–70.

[30] Khan MUK, Khan MUG, Wahab A. A Fast and Compact 3-D CNN for Hyperspectral Image Classification. IEEE Geoscience and Remote Sensing Letters. 2023;19(5):1976–87.

[31] He L, Li H, Plaza A, Bioucas-Dias JM. A novel self-paced learning framework for hyperspectral image classification. IEEE Transactions on Geoscience and Remote Sensing. 2023;62(4):1738–52.

[32] Wu TY, Li H, Kumari S, Chen CM. A Spectral Convolutional Neural Network Model Based on Adaptive Fick’s Law for Hyperspectral Image Classification. Comput Mater Continua. 2024;79(1):19–46. doi: 10.32604/cmc.2024.048347

[33] Wang J, Guan R, Gao K, Li Z, Li H, Li X, et al. Multi-level Graph Subspace Contrastive Learning for Hyperspectral Image Clustering. arXiv. 2024. doi: 10.48550/arXiv.2404.05211

[34] Chen S, Jin M, Ding J. Hyperspectral remote sensing image classification based on dense residual three-dimensional convolutional neural network. Journal of Remote Sensing. 2023;23(5):1637–51. doi: 10.11834/jrs.20231187

[35] Zhang Y, Zhang L, Du B. Hyperspectral image classification based on deep feature fusion and residual learning. IEEE Transactions on Geoscience and Remote Sensing. 2021;59(2):1576–90. doi: 10.1109/TGRS.2020.3008860

[36] Elsharkawy SM, et al. Hyperspectral Remote Sensing Images Classification Using Fully Convolutional Neural Network. IEEE Access. 2020;8(1):10404–14.

[37] Fauvel M, Chanussot J, Benediktsson JA. A spatial-spectral kernel-based approach for the classification of remote-sensing images. Pattern Recognition. 2012;45(1):381–92.

[38] Camps-Valls G, Gomez-Chova L, Munoz-Mari J, Vila-Frances J, Calpe-Maravilla J. Composite kernels for hyperspectral image classification. IEEE Geoscience and Remote Sensing Letters. 2006;3(1):93–7. doi: 10.1109/LGRS.2005.857019

[39] Bruzzone L, Carlin L. A multilevel context-based system for classification of very high spatial resolution images. IEEE Transactions on Geoscience and Remote Sensing. 2006;44(9):2587–600. doi: 10.1109/TGRS.2006.875360

