# Supplementary material for: Multi-Scale Depthwise Separable Capsule Network for hyperspectral image classification
Source: PLoS One. 2024 Aug 28;19(8):e0308789. doi: 10.1371/journal.pone.0308789 (PMC11357079; doi:10.1371/journal.pone.0308789)
Supplement: Attachment — Submitted filename: 1.docx [file pone.0308789.s002.docx]

1.The research paper lacks gaps of stud as papers cited are form year 2023 and not included from 2024

2.The graphical representation of analysis of the parameters are not done properly

3. Differentiate between equation number 4 and 5 i.e which parameters they are representing

4. The paper is not set according to the format of the journal

5.Flow of the paper is not streamlined
